# Supplementary material for: Comparison between Membrane and Thermal Dealcoholization Methods: Their Impact on the Chemical Parameters, Volatile Composition, and Sensory Characteristics of Wines
Source: Membranes (Basel). 2021 Dec 1;11(12):957. doi: 10.3390/membranes11120957 (PMC8708490; doi:10.3390/membranes11120957)
Supplement: Supplementary file 1 [file membranes-11-00957-s001.zip › membranes-1478470-supplementary.pdf]

Table S1. Odor activity values (OAV > 0.1) and odor description of the would-be impact odorants of the original wines and the dealcoholized (0.7% v/v) fractions.

Compounds	Odor description*a*	Odor threshold (μg/L)[b]	Odor activity value									Aroma classes[c]
			White wine			Rose wine			Red wine			
			CK	RO	VD	CK	RO	VD	CK	RO	VD	
Esters												
Isoamyl acetate	Banana	30	71.840^{a}	24.330^{b}	17.197^{b}	180.722^{a}	59.243^{b}	ND	37.415^{a}	15.107^{b}	5.773^{c}	2
Ethyl hexanoate	Fruity, green apple	14	153.737^{a}	13.152^{b}	0.164^{b}	122.313^{a}	9.075^{b}	0.133^{c}	69.034^{a}	11.400^{b}	0.031^{c}	2
Hexyl acetate	Fruity, herb	670	0.044^{a}	0.029^{b}	0.014c	1.727^{a}	0.292^{b}	0.128^{b}	0.107	ND	ND	2,6
Ethyl octanoate	Floral, fruity, pear	240	37.464^{a}	0.384^{b}	0.074^{b}	16.068^{a}	0.858^{b}	0.069^{c}	10.438^{a}	0.242^{b}	0.026^{c}	1,2
Ethyl decanoate	Fruity, grape	200	9.096^{a}	0.083^{b}	0.024^{b}	5.884^{a}	0.485^{b}	0.011^{c}	3.678^{a}	0.018^{b}	0.004^{b}	2
Phenethyl acetate	Floral, rose	250	0.939^{a}	0.247^{b}	0.003^{b}	2.771^{a}	1.190^{b}	0.648^{c}	0.347^{a}	0.126^{b}	ND	1
Alcohols												
1-Hexanol	Cut grass, floral	110	1.123^{a}	0.421^{b}	0.007^{b}	1.183^{a}	0.490^{b}	0.004^{c}	3.148^{a}	1.219^{b}	ND	1,6
1-Octanol	Jasmine, lemon	40	0.326^{a}	0.164^{ab}	0.012^{b}	0.177^{a}	0.110^{b}	0.006^{c}	0.788^{a}	0.320^{b}	0.007^{c}	1,3
2-Phenylethanol	Floral, rose, honey	10000	0.093^{a}	0.027^{b}	0.018^{b}	0.090^{a}	0.067^{b}	0.055^{c}	0.278^{a}	0.069^{b}	0.056^{c}	1
Dodecanol	Fatty	7	1.449^{a}	0.664^{b}	0.021^{c}	1.336^{a}	0.355^{b}	0.035^{c}	ND	ND	ND	3
Acids												
Hexanoic acid	Cheese, fatty	420	1.291^{a}	0.273^{ab}	0.244^{b}	0.985^{a}	0.255^{b}	0.216^{c}	0.925^{a}	0.192^{b}	0.095^{c}	3
Octanoic acid	Rancid, fatty	500	2.880^{a}	0.885^{b}	0.378^{b}	6.187^{a}	0.228^{c}	0.578^{b}	1.454^{a}	0.431^{b}	0.067^{c}	3
Decanoic acid	Rancid fat	1400	0.104^{a}	0.023^{b}	0.012^{b}	0.080^{a}	0.072^{b}	0.012^{c}	0.125^{a}	0.020^{b}	0.011^{c}	3
Terpenics and C13-Norisoprenoids												
Linalool	Floral	25	0.335^{b}	0.543^{a}	0.030^{c}	0.344^{a}	0.299^{a}	0.053^{b}	ND	ND	ND	1
β-damascenone	Rose, floral, fruity	0.05	737.636^{a}	411.738^{ab}	38.559^{b}	271.403^{a}	174.947^{b}	7.090^{c}	293.970^{a}	111.771^{b}	18.690^{c}	1,2
Geraniol	Rose, geranium	20	1.241^{a}	0.241^{b}	0.016^{c}	0.595^{a}	0.228^{b}	0.004^{c}	ND	ND	ND	1
Geranyl acetone	Floral	60	0.174^{a}	0.113^{a}	0.011^{b}	0.075^{a}	0.037^{b}	0.006^{c}	0.077	ND	ND	1
Others												
Benzaldehyde	Sweet, fruity	350	0.172^{a}	0.036^{b}	0.018^{b}	0.313^{a}	0.043^{b}	0.002^{b}	0.033^{a}	0.011^{b}	0.003^{c}	2

[a] Odor descriptions are mainly obtained from the following literatures: flavornet database (http://www.flavornet.org, accessed August 2021), [60,81,90–93,82–89]
[b] Thresholds are mainly gotten from the following literatures: [9,60,92,93,107–109,81,83,85–88,90,91]
[c] Each compound was attributed to 1 or more aroma class of sensory descriptors as follows: 1, floral; 2, fruity; 3, fatty; 4, pungent; 5, spicy; 6, vegetative; 7, earthy.
